# Supplementary material for: Energy Reserve Allocation in the Trade-Off between Migration and Reproduction in Fall Armyworm
Source: Insects. 2024 Oct 16;15(10):809. doi: 10.3390/insects15100809 (PMC11509284; doi:10.3390/insects15100809)
Supplement: Supplementary file 1 [file insects-15-00809-s001.zip › insects-3249688-supplementary.pdf]

Supplemental Information

Energy reserves allocation in the trade-off between migration and reproduction in fall armyworm

Chuan-Feng Xu, Peng-Cheng Liu, Jason W. Chapman, Karl R. Wotton, Guo-Jun Qi,Yu-Meng Wang, Gao Hu

Table S1 Quality of RNA-seq data.

Samples	Raw reads (M)	Clean reads (M)	Clean bases (G)	Q20 (%)	Q30 (%)	GC content (%)	Total mapped ratio (%)	Unique mapped ratio (%)
MG-II.1	42.06	41.26	6.19	96.39	91.25	42.59	91.54	87.19
MG-II.2	43.09	41.95	6.29	96.37	91.19	42.75	91.53	86.90
MG-II.3	42.11	41.32	6.20	96.36	91.17	42.93	91.57	87.43
MG-III.1	41.94	41.10	6.16	96.66	91.69	42.11	91.54	86.99
MG-III.2	42.20	41.38	6.21	96.80	92.02	41.76	91.15	86.33
MG-III.3	42.10	41.39	6.21	96.67	91.75	42.42	91.39	87.29
NMG-II.1	43.57	42.54	6.38	96.59	91.69	41.85	91.32	85.19
NMG-II.2	42.22	41.52	6.23	96.42	91.29	42.38	91.07	86.22
NMG-II.3	43.27	42.22	6.33	96.55	91.59	41.62	90.99	86.38
NMG-III.1	42.33	41.67	6.25	96.58	91.69	41.96	91.11	86.70
NMG-III.2	42.12	41.21	6.18	96.47	91.44	41.84	91.06	85.99
NMG-III.3	42.19	41.42	6.21	96.58	91.68	43.08	91.65	87.43

MG-II and MG-III means migratory females with ovarian development levels of II and III, respectively; NMG-II and NMG-III means non-migratory females with ovarian development levels of II and III.

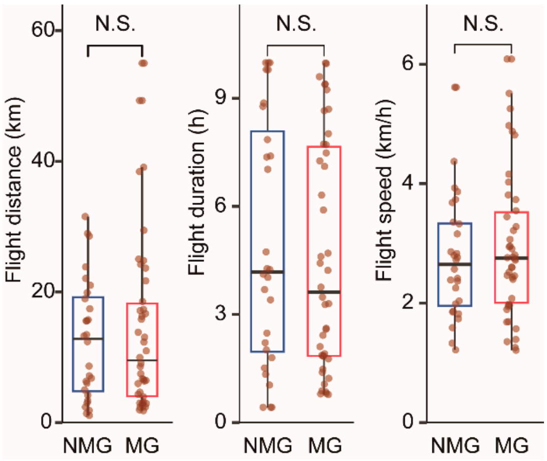

Figure S1 The flight trait divergence between migratory and non-migratory males in *S. frugiperda*. MG and NMG represent migratory and non-migratory individuals, respectively. Data are presented as max, minimum, median, upper and lower quartiles values, N.S., $P > 0.05$. Each dot represents a single individual ($n \geq 20$, Mann−Whitney U-test).

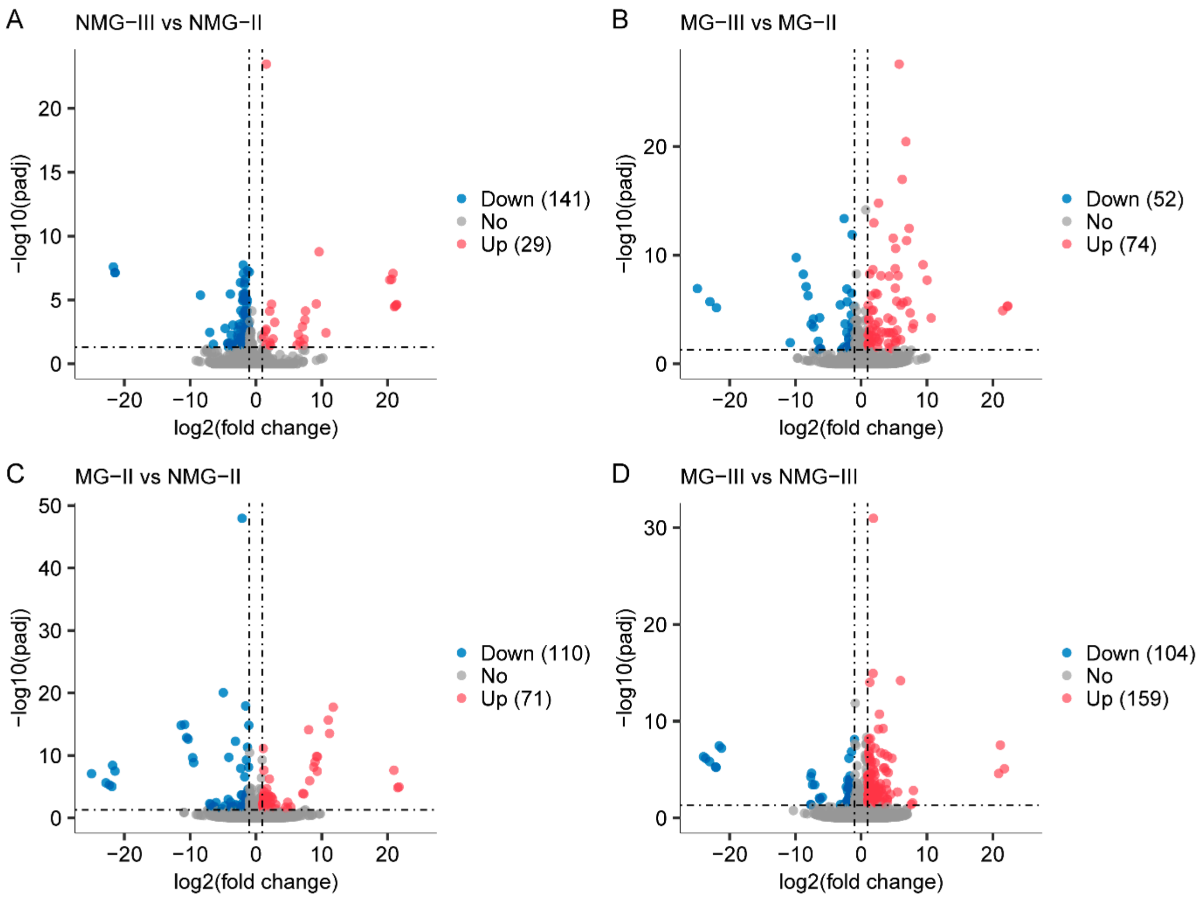

Figure S2 Volcano plot analysis of DEGs between NMG-II and NMG-III (A), MG-II and MG-III (B), NMG-II and MG-II (C), and NMG-III and MG-III (D). Up-regulated and down-regulated genes were identified based upon adjusted p-value < 0.05 and the absolute value of $\log_2$ ratio > 1.

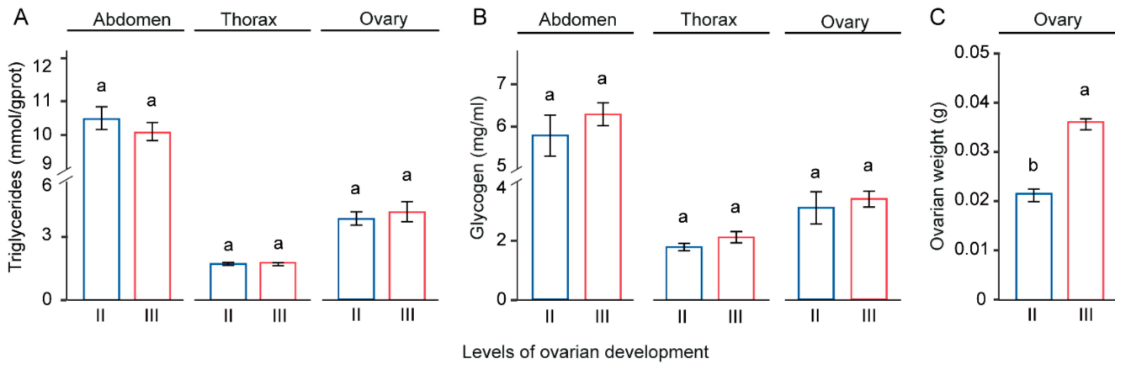

Figure S3 Analysis of energy substance content in *S. frugiperda* females with different ovarian development levels. (A and B) Comparison of triglycerides (A) and glycogen (B) levels between ovarian development levels of II and III. (C) The weight of ovaries was measured as parallel. Data were presented as mean values ± SEM, the different lowercase letters above bars indicate significant difference ($n \geq 6$, Student's t test).
